# Blood meal sources of wild and domestic *Triatoma infestans* (Hemiptera: Reduviidae) in Bolivia: connectivity between cycles of transmission of *Trypanosoma cruzi*

**DOI:** 10.1186/s13071-016-1499-0

**Published:** 2016-04-18

**Authors:** Rosio Buitrago, Marie-France Bosseno, Stéphanie Depickère, Etienne Waleckx, Renata Salas, Claudia Aliaga, Christian Barnabé, Simone Frédérique Brenière

**Affiliations:** IRD, Institut de Recherche pour le Développement, UMR INTERTRYP, (IRD-CIRAD), Interactions hôtes-vecteurs-parasites-environnement dans les maladies tropicales négligées dues aux trypanosomatidés, 911 Av. Agropolis, Montpellier, cédex 5, 34394 France; Instituto Nacional de Laboratorios de Salud (INLASA), Laboratorio de Entomología Médica, Rafael Zubieta #1889, Miraflores, Casilla M-10019 La Paz Bolivia

**Keywords:** Blood meal sources, Wild *Triatoma infestans*, Feeding habits, Chagas disease, Triatomines

## Abstract

**Background:**

Chagas disease is a major public health problem in Latin America. Its etiologic agent, *Trypanosoma cruzi*, is mainly transmitted through the contaminated faeces of blood-sucking insects called triatomines. *Triatoma infestans* is the main vector in various countries in South America and recently, several foci of wild populations of this species have been described in Bolivia and other countries. These wild populations are suspected of affecting the success of insecticide control campaigns being carried out in South America. To assess the risk that these *T. infestans* populations pose to human health, it is helpful to determine blood meal sources.

**Methods:**

In the present work, blood meals were identified in various Bolivian wild *T. infestans* populations and in three specific areas, in both wild and intra-peridomestic populations to assess the links between wild and domestic cycles of *T. cruzi* transmission. PCR-HDA and sequencing of *Cytb* gene were used to identify these blood meal sources.

**Results and discussion:**

Fourteen vertebrate species were identified as wild blood meal sources. Of those, the most prevalent species were two Andean endemic rodents, *Octodontomys gliroides* (36 %) and *Galea musteloides* (30 %), while humans were the third most prevalent source (18.7 %). Of 163 blood meals from peridomestic areas, more than half were chickens, and the others were generally domestic animals or humans. Interestingly, blood from wild animals was identified in triatomines captured in the peridomestic and domestic environment, and blood from domestic animals was found in triatomines captured in the wild, revealing links between wild and domestic cycles of *T. cruzi* transmission.

**Conclusion:**

The current study suggests that wild *T. infestans* attack humans in the wild, but is also able to bite humans in domestic settings before going back to its natural environment. These results support the risk to human health posed by wild populations of *T. infestans*.

## Background

In Bolivia and other South American countries, *Triatoma infestans* (Hemiptera: Reduviidae) is the main vector of *Trypanosoma cruzi*, the causative agent of Chagas disease [1]. *Triatoma infestans* is very well adapted to the domestic environment and is the target of large-scale campaigns of vector control based on insecticide spraying. This control strategy was initially proposed considering this species to be almost exclusively domestic, except for small wild foci primarily found in the valleys of Cochabamba in Bolivia [2–4]. However, the discovery of wild populations of *T. infestans* in other ecoregions such as Bolivian Chaco [5, 6] indicates that wild populations of *T. infestans* in Bolivia are more widely distributed than previously assumed [7–11]. They have even been reported in Argentina, Paraguay and Chile [12–14]. These wild populations could potentially pose a risk to human health. Indeed, they can move from the wilderness to human dwellings [15], where they can occasionally come into contact with humans and/or colonize the intra-peridomiciles, creating a dangerous situation of persistence of man-vector contact.

Haematophagy is an obligatory habit of triatomines. Consequently, wild populations of triatomines are closely related to food availability and therefore the distribution of mammals in nature. Surprisingly, few data exist on blood meal sources and trophic preferences (if any) of wild populations of triatomine species. For wild *T. infestans,* the only data available deal with populations captured in rocky outcrops in the high valleys of Cochabamba (Bolivia) where they were found to be associated with small rodents of the genera *Bolomys* and *Phyllotis* and marsupials of the genus *Thylamis* [7, 16]. Additionally, in the Andean valleys of La Paz (Bolivia), Buitrago et al. (2010) [8] reported the discovery of wild *T. infestans* in deep cracks serving as shelters for viscachas (*Lagidium viscacia*), suggesting that this Andean rodent could be a possible blood meal source of wild *T. infestans*, but without clear evidence. Finally, except for a short note presenting the preliminary data of the current study [17], we are not aware of any studies reporting molecular evidence of wild *T. infestans* blood meal source.

The aim of this study was to use molecular techniques to identify the blood meal sources of wild and domestic populations of *T. infestans* in Bolivia and to assess the risk that wild *T. infestans* pose to human health as well as the links between wild and domestic cycles of transmission of *T. cruzi*.

## Methods

### Study area and collection of triatomines

First, studies investigated wild *T. infestans* captured across three eco regions in the endemic area of domestic *T. infestans* in Bolivia: [18] Inter Andean Dry Forests, Prepuna, and Gran Chaco ecoregions [19]. The details of this sample consisting of 618 bugs are shown in Table 1.

**Table 1:** Geographic origin of wild *T. infestans* in three ecoregions of Bolivia

Ecoregion**	Department	Location code	Latitude (S)	Longitude (W)	Altitude (m)	No. of blood meals	
						Processed	Identified
BSIA	La Paz	SAP	16°48'47.0''	67°42'10.0''	1880	48	17
BSIA	La Paz	COS 01	16°49'54.1''	67°42'20.6''	1905	9	2
BSIA	Potosi	BSIA	18°00'43.0''	65°48'32.0''	2000	45	5
BSIA	La Paz	POO	16°51'06.8''	67°42'30.0''	2040	4	0
BSIA	La Paz	*TUN 03	16°53'12.2''	67°42'43.1''	2095	20	6
BSIA	La Paz	*QUE 01	17°01'54.8''	67°40'38.6''	2159	2	1
BSIA	La Paz	*VIZ 02	16°55'48.8''	67°41'32.9''	2182	78	8
BSIA	Cochabamba	BSIA 09	17°56'01.0''	65°23'06.5''	2182	1	0
BSIA	La Paz	*CAC 03	17°00'30.1''	67°39'25.2''	2356	72	14
BSIA	La Paz	*TUN 02	16°43'11.5''	67°52'25.7''	2427	14	1
BSIA	La Paz	*RUI 01	16°42'56.4''	67°52'13.5''	2459	7	1
BSIA	La Paz	*TUN 06	17°04'25.2''	67°37'59.7''	2493	5	3
BSIA	La Paz	BSIA 11	17°27'45.5''	66°18'51.0''	2543	3	0
BSIA	La Paz	*TUN 07	17°04'24.2''	67°38'42.7''	2543	1	1
BSIA	La Paz	*BSIA 12	17°03'34.8''	67°39'58.4''	2583	10	2
BSIA	Cochabamba	QUI	17°25'20.0''	66°17'40.0''	2600	72	20
BSIA	La Paz	*LIE 01	17°04'44.6''	67°37'57.0''	2602	17	3
BSIA	La Paz	*CAC 02	17°04'07.7''	67°39'25.5''	2645	5	1
BSIA	Cochabamba	BSIA 14	17°25'28.9''	66°15'53.0''	2689	33	7
BSIA	Cochabamba	CAC 04	17°28'37.5''	66°08'16.1''	2710	22	1
BSIA	La Paz	*TUN 01	16°42'25.4''	67°59'37.6''	2757	44	14
BSIA	La Paz	*BSIA 13	16°42'02.75''	67°59'54.9''	2765	6	1
BSIA	La Paz	*TUN 04	17°08'10.8"	67°35'17.9"	2767	5	1
BSIA	La Paz	*VIZ 01	16°41'25.4''	68°00'39.7''	2821	24	7
BSIA	La Paz	*TUN 05	17°07'32.0"	67°35'59.5"	2864	7	2
					Total BSIA	554	118
PP	Potosi	VIS	21°37'16.8''	65°48'46.0''	2963	10	7
PP	Potosi	TP 01	21°44'51.0''	65°49'26.0''	3080	34	19
					Total PP	44	26
GC	Santa Cruz	SS-2	19°25'7.39"	62°38'24.8"	412	2	0
GC	Tarija	Z 01	21°50'48.2	63°14'51.7''	443	2	0
GC	Santa Cruz	SA	20°13'42.4"	62°54'2.4"	553	7	0
GC	Santa Cruz	Z07	20°15'07.9''	62°59'10.2''	599	7	0
GC	Santa Cruz	Z08	20°11'02.6''	63°01'21.5''	614	2	0
					Total GC	20	0
					Total general	618	144

* Results of these trapping sites were published in Buitrago et al. [8]

**The ecoregions are defined according to Ibisch et al. [19]; BSIA, Inter-Andean Dry Forest; PP, Prepuna; GC, Gran Chaco

The second sample analysed was composed of peridomestic populations captured in three different localities where the above wild populations were analysed. The three localities included the rural areas of Sapini (16°48'47"S, 67°42'10" W, 1880 m) and Thago Thago (18°0'41.53"S, 65°48'32.68"W, 2000 m) and the urban periphery of the city of Quillacollo (17°25'22.03"S, 66°17'30.38"W, 2600 m). These three areas are located in the Inter-Andean Dry Forests. Sapini is located in the La Paz Department near the Luribay River. Since 2003, the village has been under entomological vigilance by the La Paz Departmental Health Service. Despite vector control based on insecticide spraying every 6 months, this village suffers constant reinfestation. It consists of 30 dwellings surrounded by sedimentary cliffs where we captured wild populations of *T. infestans.* Thago Thago is located in the Potosí Department. It is a very small village with 10 dwellings, near a road and surrounded by high mountains with rocky outcrops infested by wild *T. infestans*. Quillacollo is located 10 km from the city of Cochabamba. The area is surrounded by hills and the dwellings are very close to rocky outcrops infested by wild populations of *T. infestans*.

Before starting the entomological study, the research team met the authorities in each area to explain the purpose of the work and to obtain their agreement.

In all cases, wild triatomines were collected using mice-baited adhesive traps [20]. All triatomines caught in the same trap were put in a single tube and transported live to the laboratory. In domestic and peridomestic areas, triatomines were captured by active search and placed in a single tube per site of capture.

### Laboratory processing

The species, sex and stage of the triatomines were determined following the taxonomic keys of Lent & Wygodzinsky [21]. To determine blood meal sources and for direct assessment of the presence or absence of *T. cruzi*, abdominal contents were recovered in two ways: i) by abdominal dissection and removal of the entire digestive tract with tweezers, ii) by abdominal pressure to obtain intestinal content after cutting the terminal abdomen when insects had an abundant blood meal. Samples were stored at -20 °C until processing.

### Molecular characterization of blood meal sources

DNA extraction from intestinal contents of the digestive tracts was performed with QIAamp DNA mini kit (Qiagen, Courtaboeuf, France) according to the recommended protocol for blood samples, with minor modifications as described by Buitrago et al. (2012) [22]. The blood meal sources were identified with PCR-Heteroduplex essay (PCR-HDA). PCR amplification of a 355 bp *Cytb* fragment (PCR-*Cytb*) was achieved with the set of primers previously described in Lee et al. (2002) [23]. Amplification was performed in a thermocycler (Mastercycler, Eppendorf, Hamburg, Germany). Heteroduplex (HDA) chains subsequently formed were analysed by polyacrylamide gel electrophoresis according to Buitrago et al. (2012) [22] using human DNA as a driver.

### Sequencing of PCR products

The direct sequencing of both strands of PCR products was performed by the company Macrogen (Seoul, Korea). Sequences of both strands were aligned using Clustal-W [24] provided in BioEdit version 7.2.0 [25], and corrected in case of any discrepancy by analysing the corresponding chromatograms. Sequences were trimmed to 235–319 bp and the search for the most similar sequences was done using Blast software (http://blast.ncbi.nlm.nih.gov/Blast.cgi) in GenBank. The aim was to identify the genus and species of blood meal sources, based on the degree of sequence homology. Homology of 90 % or more allows the genus and species of the blood meal source to be identified and below 90 %, only the genus.

## Results

### Blood meal sources of wild *T. infestans*

**Fig. 1:**
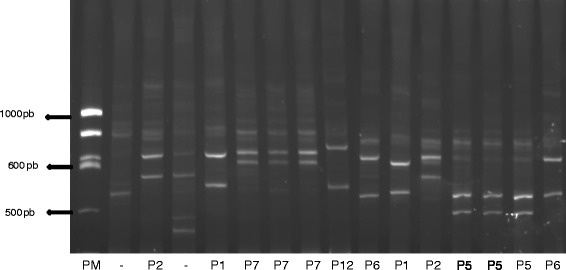
Acrylamide electrophoresis gel showing PCR-HDA patterns of DNA samples obtained from intestinal contents of wild *T. infestans*: lane 1, molecular weight; lanes 3 and 12, P2 HDA pattern (*G. musteloides*); lanes 5 and 11, P1 HDA pattern (*O.* gliroides); lanes 6, 7 and 8, P7 HDA pattern (*G. musteloides*); lane 9, P12 HDA pattern (*A. glaucinus*); lanes 10 and 16, P6 HDA pattern (*O. gliroides*); lanes 13, 14 and 15, P5 HDA pattern (*G. gallus*); lanes 2 and 4, multibanding patterns corresponding to multiple meals

The intestinal contents of 618 wild *T. infestans* captured in Andean Dry Forests, Prepuna and Gran Chaco eco regions were processed. Positive PCR (meaning that DNA from a vertebrate was present) was obtained for 371 samples, and the blood meal sources were identified for 144 samples (23.3 %) (Table 1). Multi-banding HDA patterns characteristic of multiple blood meal sources were observed for 32 other samples (8.6 %). The remaining 195 samples, including 10 from the Gran Chaco eco region, presented insufficient PCR products to obtain heteroduplex formation or for direct sequencing. The overall identified HDA patterns (144) were composed of two bands characteristic of the formation of the heteroduplex molecules. A total of 18 different heteroduplex patterns (P1 to P18) were identified. Seven of them plus two patterns of multiple banding are illustrated in Fig. 1.

To identify the species corresponding to the 18 different HDA patterns and their reproducibility, 59 PCR products were sequenced. After inference in GenBank with the Blast procedure, very high identity (98–100 %) with sequences deposited in GenBank was generally obtained, allowing identification of blood meal sources at the species level (Table 2). The sequences corresponding to identical HDA patterns always had very high identity between them (98–100 %), and they best aligned with the same species in GenBank. However, sequences corresponding to different HDA patterns could also have sequences that best aligned with the same species in GenBank. This was detected for three species: (i) *Octodontomys gliroides* had two different HDA patterns (P1 and P6) whose corresponding sequences best aligned with different GenBank sequences of *O. gliroides* (Table 2). The similarity between these sequences was 99 %; (ii) *Galea musteloides* had four different HDA patterns (P2, P7, P13 and P16) whose sequences best aligned with four different *G. musteloides* sequences deposited in GenBank. The similarity between the sequences of the patterns P16 and P7 was 96 and 99 % between P2 and P13, but between the two groups the similarity reached only 78 %; (iii) *Graomys domorum* had three different HDA patterns (P8, P9 and P11) whose sequences were best aligned with one GenBank sequence of *G. domorum*; between two of these sequences the similarity was 99 %, while with the third sequence the similarity reached only 70 %.

**Table 2:** Sequencing and identification of blood meal sources of wild *T. infestans* with different patterns of HDA

Species	HDA patterns	Size of *Cyt b* sequence	No. of sequenced PCR products	Results of the search for sequence identity in GenBank		
				Species with highest identity	GenBank: accession number	Identity (%)
Mammal	P3	315 bp	16	*Homo sapiens* (human)	AY509658.1	99
	P1	289 bp	6	*Octodontomys gliroides* (rodent)	AF370706.1	98
	P6	290 bp	6	*Octodontomys gliroides* (rodent)	GQ121127.1	99
	P2	264 bp	3	*Galea musteloides* (rodent)	GU067494.1	99–100
	P7	264 bp	3	*Galea musteloides* (rodent)	GU067530.1	100
	P13	235 bp	1	*Galea musteloides* (rodent)	GU067494.1	99
	P16	235 bp	1	*Galea musteloides* (rodent)	GU067513.1	100
	P4	255 bp	5	*Lagidium viscacia* (rodent)	AY 254887.1	99
	P8	280 bp	1	*Graomys* s.p. (rodent)	AF159291.1	88
	P9	290 bp	1	*Graomys domorum* (rodent)	AF159291.1	99
	P11	287 bp	1	*Graomys domorum* (rodent)	AF159291.1	99
	P12	319 bp	1	*Akodon glaucinus* (rodent)	KC841384.1	95
	P10	310 bp	1	*Equus asinus* (donkey)	FJ428527.1	92–99
	P14	300 bp	1	*Felis catus* (cat)	AY509646.1	99
	P15	290 bp	1	*Phyllotis wolffshoni* (rodent)	AY956698.1	98
	P17	230 bp	2	*Phyllotis xantophigus chiliensis* (rodent)	AY341053.1	96–99
Bird	P18	322 bp	1	*Subligatus modestus*	AF447623.2	100
	P5	281 bp	6	*Gallus gallus* (chicken)	EU 839454.1	100
Reptile	Absent^a^	285 bp	1	*Tropidurus* s.p. (lizard)	EF616030.1	89
	Absent^a^	294 bp	1	*Gymnodactylus* s.p. (lizard)	AY630397.1	83

^a^without profile

**Fig. 2:**
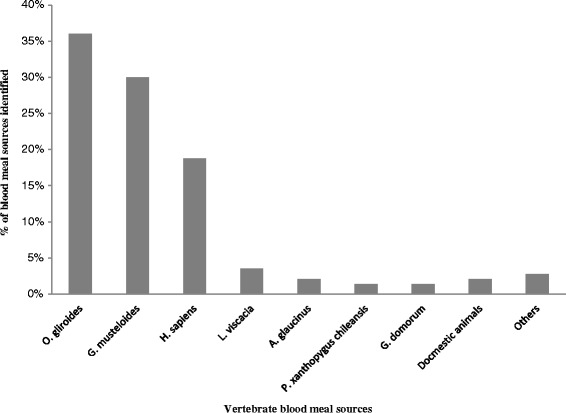
Vertebrate blood sources of wild *T. infestans* identified by PCR-HDA and sequencing

Fourteen vertebrate species were identified as blood meal sources of wild *T. infestans*: 10 mammal species, two bird species, and two reptiles (Table 2). The most prevalent blood meal was *O. gliroides* (52/144, 36.6 %), a rodent of the family Octodontidae, indigenous to the Andes (Fig. 2). Seventy-three percent of these blood meal sources were detected in 2^nd^ to 5^th^ nymphal instars of wild *T. infestans* caught in the Inter-Andean Dry Forests and the Prepuna eco regions. The second most prevalent species was *G. musteloides* (43/144, 30.5 %), a rodent of the family Caviidae. Sixty-eight percent of these blood meal sources were identified in 3^rd^ to 5^th^ nymphal instars. The third most prevalent species was *Homo sapiens* (27/144, 18.7 %), as previously detailed in Buitrago et al. (2013) [17]. Most of the human blood meals (66.7 %) were identified in 2^nd^ to 5^th^ nymphal instars captured in areas close to (50–200 m) or more distant from (~400–800 m) human habitation, in various ecotopes such as sedimentary cracks surrounded by fields of crops, prickly pear fields, sedimentary cliffs and rocky outcrops. The other identified blood meal sources included viscacha (*Lagidium viscacia*), four other rodent species, one species of wild bird and three species of domestic animals in 4^th^ nymphal instars (a donkey at 50 m from the human habitat, a cat at 120 m and a chicken at 200 m) (Table 2, Fig. 2).

### Blood meal sources of wild and intra-peridomestic *T. infestans* in three specific areas

#### Sapini

**Fig. 3:**
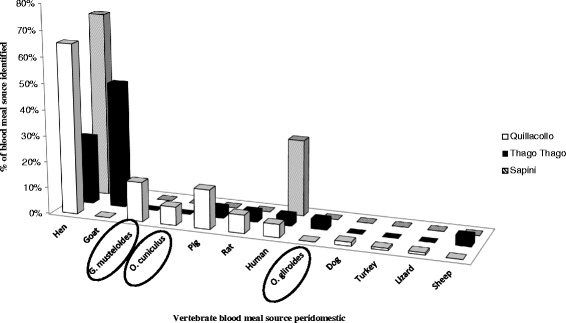
Vertebrate blood meal sources of intra-peridomestic *T. infestans* identified by PCR-HDA and sequencing in three specific areas (Quillacollo, Thago Thago and Sapini). The ellipses show the wild animals identified

In the sylvatic area around Sapini, 50 intestinal contents were processed and 17 blood meal sources (34 %) were identified. Three species of wild rodents and human were identified (Table 3). The most prevalent blood meal source identified in both adult specimens and nymphal instars was *O. gliroides* (58.8 %). The second most prevalent species was *H. sapiens* (23.5 %) found in three adult specimens and a 5^th^ nymphal instar. In peridomiciles, a total of 13 samples were processed and the blood meal sources of seven samples were identified (53.8 %). The most prevalent blood sources were chicken (71 %) and human (Table 3, Fig. 3).

**Table 3:** Identification of blood meal sources of wild and peridomestic *T. infestans* in three localities in the Inter-Andean Dry Forest ecoregion

	Locality and ecotope					
	Sapini		Thago Thago		Quillacollo	
Blood meal source	Wild	Peridomestic	Wild	Peridomestic	Wild	Peridomestic
*Akodon glaucinus* (rodent)					3	
*Canis lupus* (dog)						1
*Capra hircus* (goat)				13		
*Galea musteloides* (rodent)					14	20
*Gallus gallus* (hen)		5	1	7		81
*Graomys domorum* (rodent)			1			
*Gymnodactylus* s.p. (lizard)			1			
*Homo sapiens* (human)	4	2	1	1	2	7
*Leiolopisma* s.p. (lizard)						1
*Maleagris gallopavo* (poultry)						1
*Octodontomys gliroides* (rodent)	10			1		
*Oryctolagus cuniculus* (rabbit)				2		
*Ovis aries* (sheep)				1		
*Phyllotis wolffsohni* (rodent)					1	
*Phyllotis xanthopygus chileansis* (rodent)	2					
*Rattus rattus* (rodent)				1		9
*Sublegatus modestus* (bird)	1					
*Sus scrofa* (pig)				1		9
*Tropidurus* s.p. (lizard)			1			
Total	17	7	5	27	20	129

#### Thago Thago

From the sample collected in the wilderness of Thago Thago, 45 intestinal contents were processed but only five blood meal sources (11 %) were successfully identified: a species of wild rodent, human, chicken, and two lizards were found in 2^nd^ to 4^th^ nymphal instars (Table 3). In peridomiciles, a total of 71 samples were processed and 27 blood meal sources were identified (38 %): three species of domestic animals, two species of wild animals, asynanthropic species of rodent and humans. The two most prevalent species were goat (48 %) and chickens (26 %) raised by the inhabitants of the area. It is worth noting that two species of wild animals were also identified: *O. gliroides* found in one *T. infestans* adult and *Oryctolagus cuniculus* in 3^rd^ and 4^th^ nymphal instars (Table 3, Fig. 3).

#### Quillacollo

From the sample collected in the wilderness of Quillacollo, 72 intestinal contents were processed and 20 blood meal sources (27.8 %) were identified. Three species of wild rodents and human were identified in adults and nymphal instars (Table 3). The most prevalent species was *G. musteloides*, which represented 70 % of the blood meal sources, identified in three adults and two nymphal instars. In peridomiciles, a total of 196 samples were processed and 130 blood meal sources (66 %) were identified. The blood meal sources identified were three species of domestic animals, one species of wild animal, one species of synanthropic rodent, one lizard and human. The most prevalent species was chicken (41 %). The wild rodent *G. musteloides* accounted for 10 % of the identified blood meal sources, and *Rattus rattus*, 4.5 % (Table 3, Fig. 3).

## Discussion

### Wild areas

This study (together with the preliminary data we recently published, see [17, 22]), is the first to study the molecular identification of blood meal sources of wild populations of *T. infestans*. The results indicate that wild *T. infestans* pose an epidemiological threat to humans in Bolivia since human blood was identified in 18.7 % of the bugs tested, and also suggest the occurrence of two phenomena: (i) displacement of *T. infestans* from the human habitat to the wild after feeding, and (ii) exposure of humans to bites of *T. infestans* in the wild during their regular activities. Furthermore, the discovery of three wild 4^th^ nymphal instars of *T. infestans* with blood from domestic animals (donkey, cat, and chicken at 50, 120, and 200 m, from the closer peridomicile, respectively) suggests that wild *T. infestans* feed on domestic animals when these animals circulate in close wild environment. As previously shown through population genetics of *T. infestans* [15], the present observations support the connectivity between wild and domestic cycles and it constitutes a danger to human health with infection rates by *T. cruzi* reported from 3.8 % in first nymphal instar to 85.7 % in adult insects [8]. Other wild triatomine species have been found with human blood meal, such as *Mepraia spinolai* captured in a suburban neighbourhood of Santiago de Chile [26]. Thirty-eight percent of wild *Triatoma rubida* and *Triatoma protracta* captured in hills in the US were also found to have fed on humans [27]. In Colombia, human blood meals were identified in the strictly wild species *Eratyrus cuspidatus*. Recently, a mixture of human and opossum blood was identified in wild *Triatoma dimidiata* and a mixture of human and chicken blood was identified in *Rhodnius pallencens* captured in palm trees [28].

Fourteen different vertebrate blood sources, including reptiles, were identified in wild *T. infestans*. This supports the theory of opportunistic feeding habits for *T. infestans*, as already observed in many species [29, 30]. In general, it was found that wild *T. infestans* had little intestinal content, and the percentage of identification was low because the blood source DNAs were probably degraded. Together these results may suggest that in the wilderness of the study area, there is little availability of food, forcing triatomines to search for multiple sources of food. The low blood host availability in the wilderness might also explain the identification of reptiles as blood sources of wild *T. infestans*. Indeed, a study carried out on food preferences in three triatomine species including *T. infestans* in laboratory conditions showed that this species seems more attracted to warm-blooded animals [31]. Nevertheless, even if the low accessibility of blood sources in the wilderness could explain the identification of reptiles as blood sources, this hypothesis is not well supported if we take into account results reported in other works performed with domesticated populations of triatomines. These results reported frequent multiple blood meals [32] and a species of frog (cold blood vertebrate) was also reported as the main feeding source [30].

In the current study, the proportion of multiple blood meals (8.6 %) in the wild triatomines was not that high compared with other studies. This could be explained by low accessibility to blood sources in the wilderness as already mentioned, which means scarcity of mammals in this area. Also this can be due to the low diversity of the mammal species, but unfortunately data on species and abundance of mammals in this region are not available. Another explanation can be the technique used, the current one could be less sensitive than the immuno diffusion test used before for *T. longipennis* populations [32, 33] and cloning followed by a recently developed sequencing technique [27, 30].

The predominant blood meal source identified is *O. gliroides,* a rodent endemic to the Andes. This rodent has been found to be infected with *T. cruzi* in a study northwest of Argentina, in Pucara, an archaeological area currently uninhabited close to Jujuy [34]. This suggests that this species could be a reservoir of *T. cruzi* and that it is an important species for the development of wild colonies of *T. infestans* in Bolivia.

*G. musteloides* is the second most important blood meal source identified as sustaining wild *T. infestans*. In 2006 and 2007 in a study in Quillacollo (Cochabamba, Bolivia), this mammal was associated with wild populations of *T. infestans* [7, 16]. Recently, Torrico et al. (2013) [35], conducted a study to isolate kinetoplastid protozoan from wild mammals in three departments of Bolivia (Cochabamba, Santa Cruz and Potosí). They found nine specimens of *G. musteloides* infected with *T. cruzi* in Cochabamba; they also found one infected specimen of *G. domorum*, a species that also constitutes a food source for wild *T. infestans*.

Interestingly, *L. viscacia* (vizcacha) was also identified as a blood meal source, confirming previous suggestions [8]. This small Andean mammal that looks similar to a rabbit is well-accustomed to rocky habitats. With regards to intraspecific variability found by PCR-HDA in *G. musteloides* where more than 10 % sequence divergence was observed, as previously discussed, the presence of different species and not intraspecific variation is more likely [22]. In fact there is still no taxonomic consensus within the genus *Galea* [36].

### Specific inhabited areas and surroundings

The simultaneous analysis of the blood meal sources of triatomines collected in the wild and in the corresponding peridomestic areas provided valuable information on the behaviour of insect vectors in the context of vector-host interactions. Populations of wild triatomines are generally found to feed on a variety of wild mammals while resident species are found to feed on domestic animals. These assertions are widely supported by studies reporting chickens, humans, dogs and cats as the main blood meal sources of non-wild triatomines, including *T. infestans*, *T. sordida*, *T. longipennis* and *T. dimidiata* [5, 32, 33, 37, 38]. Our data largely confirmed these observations. Chickens were identified as the main blood meal sources in Sapini and Quillacollo and goats in Thago Thago where breeding goats is common. The blood meal sources identified generally corresponded to animals we observed occupying the capture sites. However, we must also emphasize that wild animals (*O. gliroides*, *O. cuniculus* and *G. musteloides*) were also identified as blood sources for the triatomines collected in the peridomiciles of Quillacollo and Thago Thago. These blood meal sources were identified mainly in 3^rd^ to 5^th^ nymphal instars (72.7 %) and therefore less likely to spread into the wild and return to the peridomicile. Consequently, in this specific case, these data suggest the movement of wild animals to the peridomicile. Similarly, both phenomena possibly occur at the same time. These animals circulating among wild and domestic habitats play a particular epidemiological role because they link the sylvatic cycle of the parasite with a domestic one.

In the same way, synanthropic animals such as rats also constituted an important blood meal source in the peridomiciliary area. In Quillacollo and Thago Thago, *R. rattus* represented 7 and 4 % of the identified blood meal sources, respectively. Similarly, in a study in Mexico, *R. rattus* has been reported as the main blood meal source of *Triatoma longipennis* in peridomiciliary areas [32]. As an important blood meal source for triatomines, these synanthropic animals contribute to maintaining large triatomine populations near human dwellings.

## Conclusion

In Bolivia and other South American countries, *T. infestans* is the target of large-scale campaigns of vector control based on insecticide spraying. The current study suggests that wild *T. infestans* attacks humans in the wild, but is also able to bite humans in domestic settings before going back to its natural environment. The link between the wild and domestic cycles of *T. cruzi* transmission is a danger to human health. The persistent re-infestation of houses by this species in the Andean region can be attributed, at least in part, to the dispersal of bugs from sylvatic populations, this statement is supported by genetic studies showing an absence of significant genetic differentiation between domestic populations and nearby sylvatic ones [15]. Together with the former study, our study suggests that wild populations of *T. infestans* are hindering control efforts in Bolivia and need to be taken into account in vector control strategies. While insecticide spraying may be effective for the control of triatomine populations that are well established inside dwellings, the dispersal of *T. infestans* from the wild environment makes vector control challenging. Consequently, for the control of *T. infestans*, the use of other strategies (such as the use of window screens) together with insecticide spraying will be needed in areas where wild populations of this species exist [39, 40].
